# Staged definitive repair for pulmonary atresia and ventricular septal defect 40 years after palliative surgery: a case report

**DOI:** 10.1186/s44215-023-00049-y

**Published:** 2023-06-05

**Authors:** Noriyoshi Ebuoka, Norihiro Ando, Hidetsugu Asai, Nobuyasu Kato, Tsuyoshi Tachibana, Satoru Wakasa

**Affiliations:** 1 Hokkaido Medical Center for Child Health and Rehabilitation, Pediatric Cardiovascular Surgery, Sapporo, Hokkaido Japan; 2 Department of Cardiovascular Surgery, Hokkaido University Hospital, Sapporo, Hokkaido Japan

**Keywords:** Pulmonary atresia, Ventricular septal defect, Palliative surgery, Case report

## Abstract

We present a very rare case of pulmonary atresia and ventricular septal defect with staged definitive repair more than 40 years after palliative surgery. The patient, a 43-year-old male, had undergone a Waterston operation at the age of one and had been untreated since then. Two years ago, he underwent an urgent surgery for impending rupture of a huge pulmonary artery aneurysm. Then, after evaluation of cardiac and pulmonary functions, a definitive repair was performed concomitantly with aortic root replacement for the dilated aortic root. He was discharged uneventfully and received ambulatory care 5 years after surgery.

## Introduction

In recent years, the number of adults with congenital heart disease (ACHD) after definitive repair has increased. However, many patients need additional surgical treatment for specific long-term complications, such as pulmonary regurgitation after tetralogy of Fallot (TOF); such surgeries are associated with significant risks [1]. For ACHD, patients who have undergone palliative procedures and reach adulthood without definitive repair are very rare. They often require additional surgeries that can have more risk than those who have undergone definitive repair. However, the outcomes of such surgeries remain unknown due to the very few reports regarding such conditions. In addition, definitive repair for these patients in adulthood has generally not been viewed as feasible. Herein, we report a patient who underwent surgery to mitigate long-term complications after a childhood palliative procedure to treat pulmonary atresia (PA) and ventricular septal defect (VSD), which was definitively repaired more than 40 years later.

## Case description

**Fig. 1:**
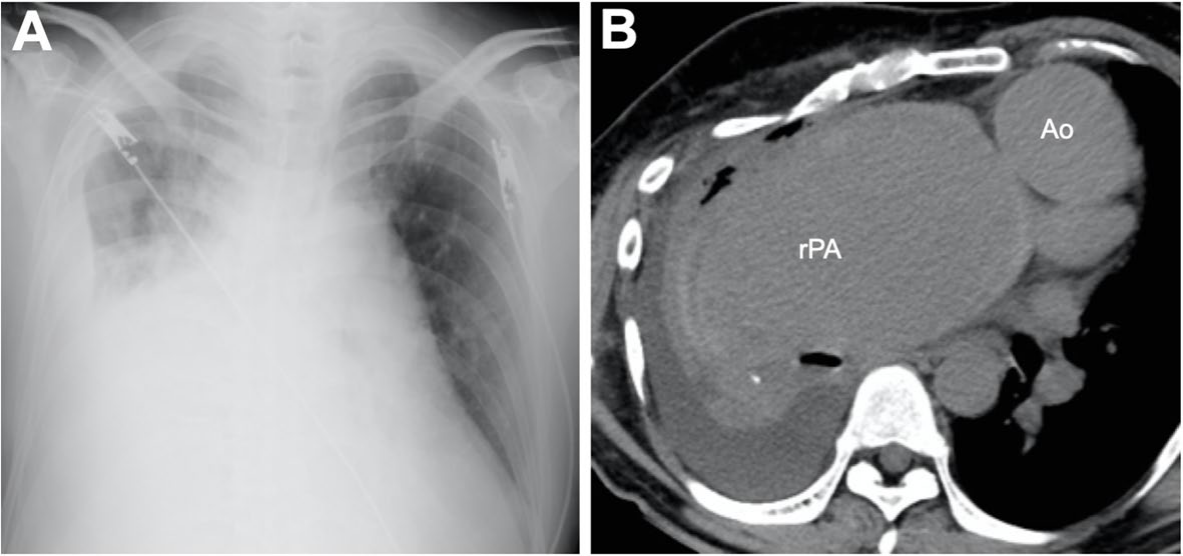
Impending rupture of pulmonary artery aneurysm. Large right pulmonary artery aneurysm and pulmonary effusion identified on **A** chest roentgenogram and **B** computed tomography. Ao, ascending aorta; rPA, right pulmonary artery

A 43-year-old man had undergone a Waterston operation for PA/VSD at age 1, as a more definitive surgery was considered unviable at that time. He was not available for postsurgical follow-up and had been engaged in physical labor since his high-school graduation without any exercise restrictions. Four years prior, he had been hospitalized for pneumonia. Computed tomography (CT) revealed a right pulmonary artery aneurysm (PAA) with a maximum diameter of 75 mm. Two years later, he was referred to our hospital with suspicion of an impending PAA rupture, because he developed dyspnea on exertion. CT revealed an enlargement of the right PAA (95 mm) with a right pleural effusion, upon drainage, 800 ml of slightly bloody was collected (Fig. 1A, B). He presented with severe hypoxemia (a saturation of percutaneous oxygen (SpO2) was 78% at 10 l/min oxygen) and difficulty of breathing. Echocardiography revealed that both ventricle had normal function and balanced volumes without atrioventricular valve regurgitation; however, cardiac catheterization was not performed due to the risk of rupture.

**Fig. 2:**
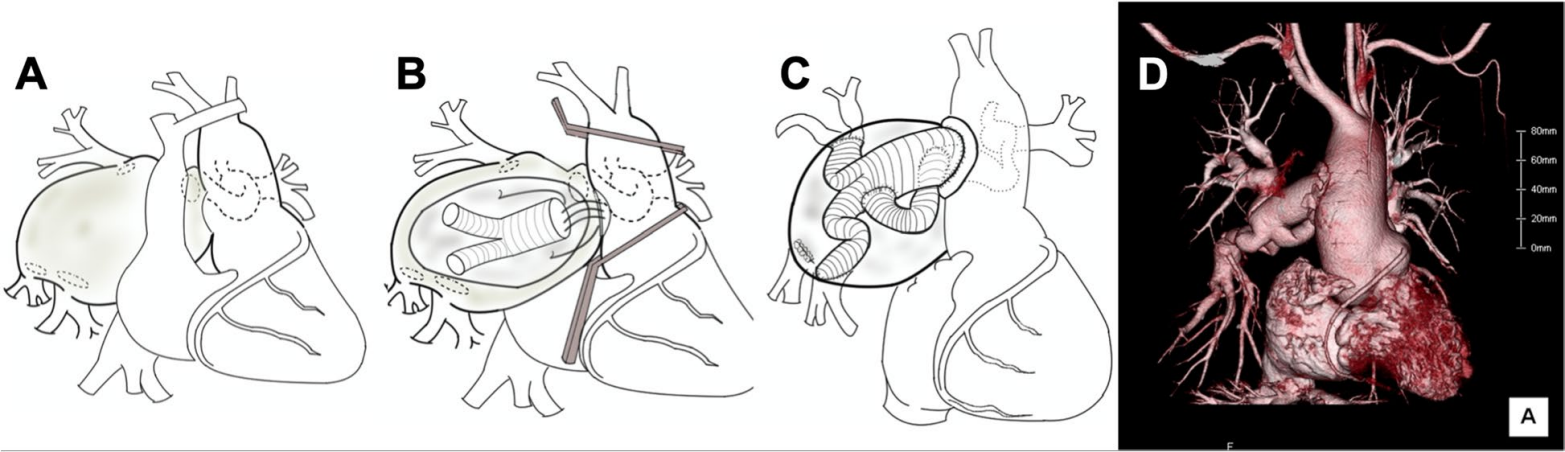
Repair schematics. **A** Dilated right pulmonary artery. **B**, **C** Aneurysm replacement using a Y-shaped vascular prosthesis, anastomosed on the **B** Waterston anastomosis site and **C** pulmonary artery branches, including the left pulmonary artery. **D** Postoperative CT image showing patency of all anastomoses

The patient underwent emergency surgery for the PAA. Resection surgery was performed over aneurysmorrhaphy because no PAA rupture point was found in preoperative CT. Operative schemas are shown in Fig. 2. We established extracorporeal circulation via the femoral artery and vein before sternal opening to prepare for any intra-operative bleeding. A cardiopulmonary bypass of 3.0 L min^−1^ m^−2^ was needed to maintain blood pressure. The ascending aorta was clamped above and below the Waterston anastomosis site (Fig. 2B). After inducing cardiac arrest, the aneurysm was opened and the ostia of the pulmonary lobe branches of the right pulmonary artery were identified after using suctions to control bleeding from distal pulmonary arteries. There was no evidence of PAA rupture. After the main body of a Y-shaped vascular prosthesis (24 × 12 mm Hemashield Gold™ Microvel Kitted Double Velour Vascular Grafts, Maquet, Lastatt, Germany), which was similar in size to Waterston anastomosis as measured in preoperative CT images, was anastomosed on the ascending aorta, the ascending aorta was de-clamped. The prosthesis legs were then anastomosed with an inclusion technique to the left pulmonary artery, as well as the upper and lower lobar branches of the right pulmonary artery, but not the middle lobar branch due to severe adhesion. The operative, cardiopulmonary bypass, and arrest times were 766, 464, and 79 min, respectively. Postoperative CT revealed patency of all anastomoses (Fig. 2D). The postoperative course was uneventful, and the patient was discharged 38 days after surgery, and followed up as an outpatient without definitive repair since he was busy with work.

Two years after the operation, dilatation of the aortic root (from 50 to 57 mm in 2 years), which had three even cusps, was identified on CT. Cyanosis remained significant, with SpO_2_ of 88% in room air, while cardiac function was within the normal range. A catheter examination demonstrated high pulmonary blood flow (Qp/Qs, 2.7), mean pulmonary blood pressure (65 mmHg), and pulmonary capillary wedge pressure (13 mmHg) with a tolerable pulmonary vascular resistance (4.5 units/m^2^).

**Fig. 3:**
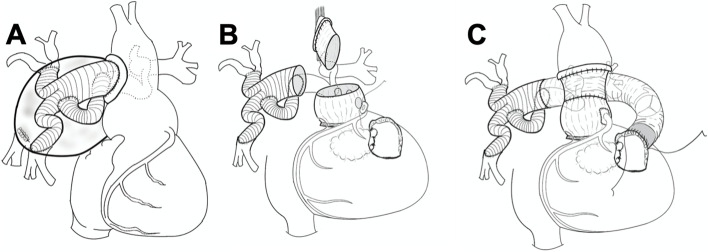
Schematics of definitive repair with aortic root replacement. **A** Dilated aortic root. **B** Aortic root replacement, preserving the aortic valve. **C** Right ventricular outflow tract reconstructed using a composite graft

Thus, we decided to perform an aortic root replacement with a definitive repair of PA/VSD (Fig. 3). After cardiopulmonary bypass with moderate hypothermia commenced through the femoral artery and vein, a valve-sparing aortic root replacement using a 28-mm prosthesis (Gelweave™ Valsalva, Terumo corporation, Tokyo, Japan) was performed by only confirming that the three free margins were equal as preoperative measurements (Fig. 3B).

Then, the VSD was closed using a 0.4-mm-thick, expanded polytetrafluoroethylene membrane (W. L. Gore & Associates, Inc., Newark, DE). Right ventricular outflow tract (RVOT) reconstruction was performed using a 26-mm Triplex composite graft (Terumo Corporation, Tokyo, Japan) and a bioprosthetic valve (Epic Supra Aortic Valve, Abbott, Chicago, USA).

In the RVOT reconstruction, distal anastomosis of the composite graft was placed on the prosthesis that had been used in the previous surgery for the PAA repair (Fig. 3C). The operative, cardiopulmonary bypass, and arrest times were 844, 538, and 265 min, respectively. Postoperative echocardiography showed mild aortic regurgitation; no residual shunt or RVOT stenosis was identified. A contrast-enhanced CT was not performed due to renal dysfunction. The patient was discharged on postoperative day 34. Five years after surgery, latest echocardiography showed mild-to-moderate aortic regurgitation without RVOT stenosis; there were no heart failure symptoms.

## Comments

This is a very rare case of successful repair of PAA with impending rupture and completion of definitive repair in adulthood, decades after a Waterston operation. In the literature, it has been reported to be difficult to save patients’ lives in such conditions. Hull et al. reported two cases of a ruptured PAA after Waterston shunt, however, the patients died in adulthood due to rupture of true PAA [2]. Oosterhof et al. reported a patient who reached adulthood after the Potts shunt for TOF, but a definitive repair could not be performed due to unacceptably high operative risk [3]. Thus, carefully selected patients can successfully undergo such procedures. We considered that preservation of ventricular function and good pulmonary condition, even under longstanding abnormal pulmonary blood flow after palliative surgery, would play a key role in the success of definitive repair in our patient. Therefore, we carefully maintain the SpO2 to < 90% on an outpatient basis. Vaikunth et al. [4] reported that ACHD is generally associated with heart failure with reduced ejection fraction, although Anna et al. [5] reported that patients with TOF with preserved ejection fractions > 40% had better prognosis. Brida et al. [6] reported that large systemic-to-pulmonary shunts can cause pulmonary artery hypertension, which may be associated with increased morbidity and mortality, although the presence of lower SpO_2_ at rest (85–90%) and no exercise restriction would predict a better prognosis. The latter was consistent with our patient, in whom pulmonary vasculature would have been protected because pulmonary blood flow would be regulated due to moderate pulmonary stenosis at the peripheral branches caused by compression by the huge PAA. For the first PAA repair, although there was the possibility that the graft size was too large and that right ventricle-pulmonary shunt was advantageous for pulmonary perfusion, we considered that it was important to maintain the similar hemodynamics after surgery because the patient was performing manual labor with no exercise restriction. During the second surgery that included definitive repair, although the procedure for dilated aortic root was controversial since there were other method such as Bio-Bentall, we considered that a valve-sparing aortic root replacement was best for the patient due to patient’s age and avoiding aortic valve reoperation, since patients with ACHD were at high risk for reoperation regardless of the type of bioprosthetic valves used [7].

Moreover, performing PAA repair and definitive surgery in sequence likely reduces invasiveness and operative risk; in fact, both procedures had long operative times (766 and 844 min).

Although providing ACHD patients with palliative procedures alone would be associated with a high operative risk, definitive repair can be completed if both ventricular function and pulmonary condition are well maintained. Moreover, in cases requiring additional procedures for long-term complications, staged surgery should be considered to reduce operative risk.

## Data Availability

The datasets used and analyzed during the current study are available from the corresponding author on reasonable request.

## Abbreviations

ACHD: Adult congenital heart disease
PA: Pulmonary atresia
VSD: Ventricular septal defect
PAA: Pulmonary artery aneurysm
CT: Computed tomography
sPO_2_: Saturation of percutaneous oxygen
RVOT: Right ventricular outflow tract
